# Implementing a general practitioner-to-general physician eConsult service (eConsultant) in Australia

**DOI:** 10.1186/s12913-022-08663-2

**Published:** 2022-10-24

**Authors:** Jennifer Job, Caroline Nicholson, Zoe Calleja, Claire Jackson, Maria Donald

**Affiliations:** 1 UQ-MRI Centre for Health System Reform and Integration, Mater Research Institute, The University of Queensland, Level 8, Health Sciences Building, Royal Brisbane and Women’s Hospital Campus, Brisbane, QLD 4029 Australia; 2 Integrated Care and Innovation Translation, Mater Misericordiae Ltd, Brisbane, Australia; 3 Primary Care Faculty of Medicine, The University of Queensland, Brisbane, Australia; 4 General Practice Clinical Unit, Faculty of Medicine, The University of Queensland, Brisbane, Australia

**Keywords:** eConsult, Electronic consultation: general practice, General physician, Implementation

## Abstract

**Background:**

In response to lengthy wait times for specialist outpatient appointments, electronic consultation (eConsult) services have developed globally, providing asynchronous, secure and timely communication between general practitioner (GP) and specialist. This study aims to track adoption of a Queensland eConsultant service in two Australian Primary Health Networks (Western Queensland and Brisbane South) to understand key barriers and enablers to adoption and inform modification of the implementation strategy.

**Methods:**

Our theory-informed mixed-methods evaluation assessed implementation between July 2020 and March 2022. Adoption and implementation activities were prospectively recorded in bespoke tracking spreadsheets with implementation activities coded against the Expert Recommendations for Implementing Change (ERIC) strategies. Semi-structured interviews with GPs and stakeholders informed by the Consolidated Framework for Implementation Research (CFIR) were conducted to understand determinants of implementation.

**Results:**

Of the 40 practices invited to take part in the eConsultant service, 20 (50%) enrolled. Of the 97 GPs who consented, 38 sent at least one Request for Advice (RFA) to the eConsultant with a total of 112 RFA sent. Implementation was predominantly guided by eight strategies. Qualitative interviews were conducted with 11 GPs and 4 stakeholders (12 from rural/remote regions, 11 females and two sole practitioners). Interviewees felt the eConsultant service supported outpatient appointment avoidance and provided efficient, timely access to specialist support for GPs and their patients. Barriers identified to using eConsultant related to digital infrastructure, competing priorities, and keeping the service ‘front of mind’. Key enablers identified were the relative advantage of eConsultant over other options, patient benefits and COVD-19 facilitating the use of digital technology.

**Conclusions:**

This evaluation highlighted service enablers as well as user priorities for broader implementation. A focus on a well-integrated digital system and availability of a variety of eConsultant specialties are seen as key strategies to embedding the eConsultant option in GP advice processes in Australia.

**Supplementary Information:**

The online version contains supplementary material available at 10.1186/s12913-022-08663-2.

## Background

With the rise in chronic disease prevalence and increasing demand for health care services worldwide, patients are experiencing excessive delays for specialist input [1]. During excessive wait times a patient’s health deteriorates, increasing the likelihood of avoidable hospital attendance and poor health outcomes [2]. Difficulties in accessing specialist support is particularly challenging for patients in rural settings, who often have to travel long distances for specialist appointments with concomitant time and financial costs [3]. In response to these issues, electronic consultation (eConsult) services have developed which provide specialist input for general practitioners (GPs) via asynchronous, secure communication for the purpose of providing guidance to patient care [4–9]. Reduced wait times for specialist input and avoidance of face-to-face hospital outpatient visits are established outcomes of the eConsult approach in both rural and urban areas [10–13]. Well accepted by both primary care and specialist providers eConsults facilitate decision support for GPs [14, 15], and provide them with education opportunities through case-based learning [16–20]. Research has demonstrated patients appreciate the improved access to specialist input and feel the service strengthens the role of the GP in their care [21].

In 2018 we introduced an eConsult (eConsultant service) proof-of-concept in one urban general practice in Queensland, Australia. Subsequently the service was introduced to four rural practices as part of a pilot study in 2019 [22], with staged implementation to additional practices across Queensland since 2020. Our eConsultant service provides general medicine specialist support from the Mater Hospital Brisbane to participating practices state-wide. International evidence has informed program development [23] and, using our co-creation approach [24, 25], service delivery partners namely the State Health Department and relevant Australian Primary Health Networks (PHNs) have been heavily involved in all phases. PHNs are independent organisations funded by the Australian Government to address local health need, minimise gaps and duplication, and work closely with general practices to deliver high quality care [26].

While eConsultant is a relatively simple clinical intervention, implementing the service requires change in well-established primary-secondary care referral practices, and design of scalable, secure asynchronous communication processes between general practice and hospital clinical information systems. Despite secure messaging being identified as a high priority for action in Australia’s National Digital Health Strategy 2018–2022 [27] a national standardised approach continues to hamper implementation of a scalable eConsultant solution. The aim of this study is to track adoption of the eConsultant service to date to inform implementation strategy modification for expansion to other regions and specialties.

## Methods

### Design

We used a theory-informed mixed-methods evaluation to achieve the study aim. eConsultant adoption and implementation activities were recorded prospectively in bespoke tracking spreadsheets. Semi-structured interviews were conducted with GPs and stakeholders to understand determinants.

### The intervention

Our eConsultant service [22] is based on a Canadian model [4]. Currently, the service provides general medicine physician support to GPs, for patients requiring specialist input who would have otherwise been referred for an outpatient department (OPD) appointment. The general physician eConsultant accepts RFA relating to all adult physician subspecialty areas defined by the RACP except dermatology [28]. This does not include direct referral / assessment for procedures. Complex cases are in scope for GPs using the service but RFA are limited to physician advice only – case conferencing is preferred for multidisciplinary assessment and care. The eConsultant is required to respond within three business days. GPs use a template to send a Request for Advice (RFA), which must include a specific question/s, to the physician (the eConsultant), with supporting information auto-populated from the patient’s record via the GP’s clinical information system. The eConsultant replies with an answer to the problem; a request for further information; or, a recommendation that the patient is referred for a traditional OPD appointment. A documented record of the eConsultant advice is provided to the GP and all treatment decisions are made in partnership with the patient, and on the understanding that there is the option for a usual care specialist referral. The GP has the option to send additional follow-up RFAs about the same patient. GPs use the same billing practice as they would for a regular consultation, and the service is funded by the Queensland eConsultant Partnership Program (QePP) at the specialist’s current sessional rate. The research team has partnered with one rural/remote PHN—the Western Queensland Primary Health Network (WQPHN), and one urban PHN—the Brisbane South Primary Health Network (BSPHN), to implement the eConsultant service. The study eConsultant is based at the Mater Hospital Brisbane (Mater).

### Underpinning implementation theory

**Fig. 1:**
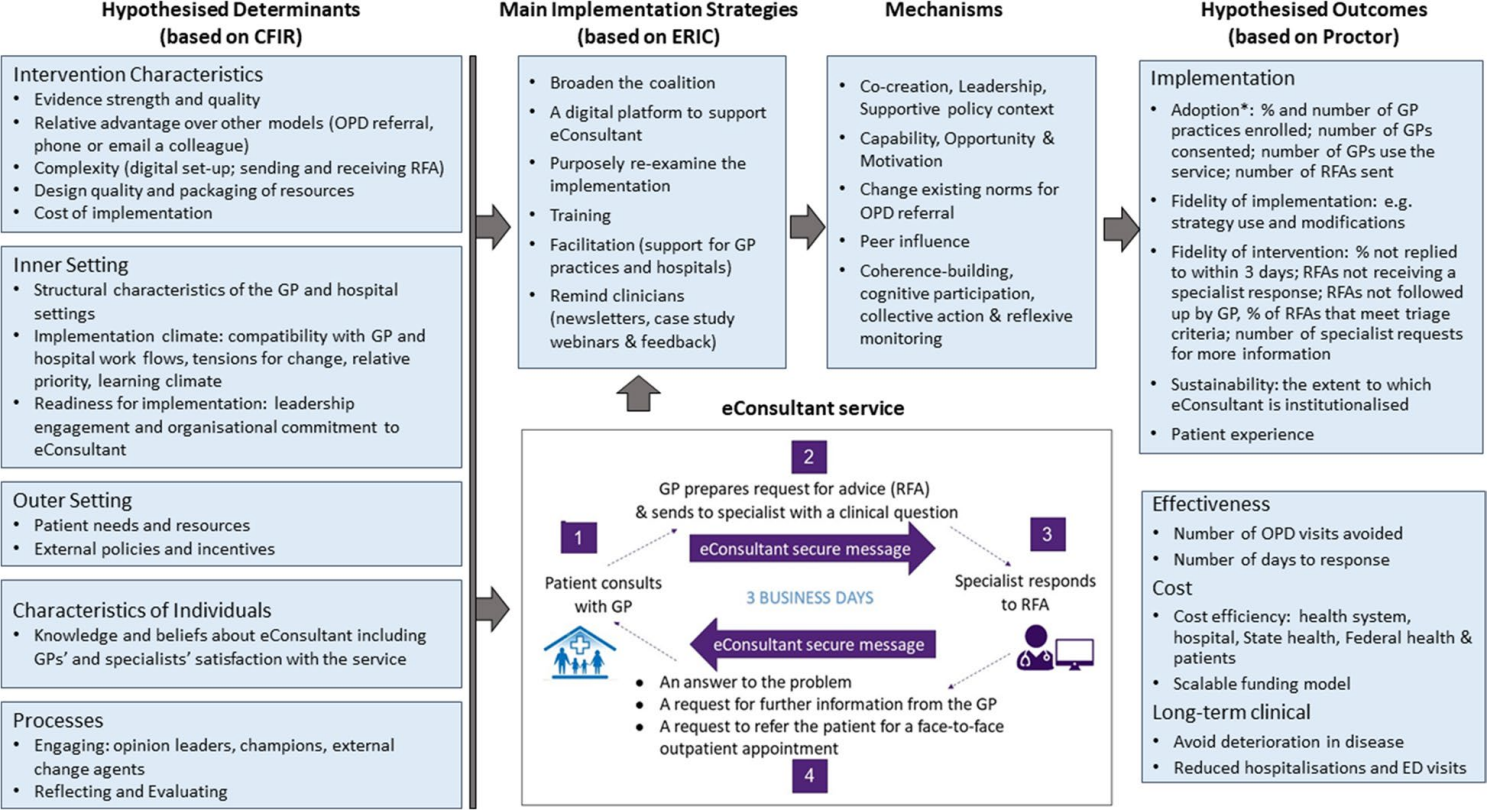
eConsultant Implementation Research Logic Model [29]. (*Adoption is the primary implementation outcome for this study)

Our research uses the Implementation Research Logic Model (IRLM) to integrate implementation theories and models that underpin our implementation (see Fig. 1) [29]. The Consolidated Framework for Implementation Research (CFIR) guides our assessment of implementation determinants (barriers and enablers) [30]. CFIR allows us to optimise our likelihood of affecting change through identification and resolution of modifiable barriers, and enhancement of identified enablers. Second, we use the Expert Recommendations for Implementing Change (ERIC) nomenclature to select implementation strategies that are scalable with stakeholders. Potential mechanisms for these strategies are proposed [31]. Finally, Proctor and colleagues’ taxonomy guides our assessment of implementation outcomes [32]. Our focus for this early phase is to explore determinants of implementation, and implementation strategy use to understand adoption of our eConsultant service.

### Data sources and measures

#### Implementation and adoption and data

From July 2020 implementation activities and adoption outcomes were recorded prospectively by the implementation researcher (JJ) in bespoke excel spreadsheets. The data included 1) indicators of adoption, and 2) an implementation activity register (date, activity, actors, and outcomes). Five measures of adoption were defined, including: the number of general practices enrolled (i.e., consented, received the training package, installed the template, and sent a successful test RFA); the proportion of general practices enrolled (i.e., number invited divided by number enrolled); the number of GPs consented (returned signed consent form); the number of GPs who have used the service (i.e., sent at least one RFA); and the number of RFAs sent (de-identified data supplied to the research team by Mater).

#### Qualitative interviews

A sample of GPs and other stakeholders (from PHNs and Queensland Health) was recruited. GPs were recruited purposively to include low (sent one RFA), medium (sent 2–7 RFA), and high-volume (sent > 7 RFA) users of the service from both rural and urban settings. A convenience sample of other stakeholders was recruited. Invitations were emailed to participants, with information describing the rationale for the research provided via an information sheet. All participants gave informed, written consent to participate and were sent the interview questions in advance, allowing them time to consider their viewpoints. Interviews were conducted between July 2021 and March 2022.

Interviews were semi-structured and informed by the CFIR [30]. The research team rated the relevance of each CFIR determinant to eConsulant on a 5-point Likert scale and those with the highest rating (mean of 4 or more) were used to determine interview questions (Fig. 1) [33]. Interview questions were developed using the Interview Guide Tool available via the CFIR website [34]. Our interview guide is provided in Additional File 1. All interviews were conducted via telephone by two researchers, one with extensive experience in qualitative research (MD) and another early career researcher trained and supervised in qualitative interviewing (ZC). Interviews continued until data saturation occurred; with no new ideas identified in responses. Interviewers recorded field notes during and after interviews. All interviews were audio-recorded, transcribed verbatim using Otter Artificial Intelligence (https://otter.ai/), cross checked against the recording (JJ), and anonymised.

### Data analysis

#### Implementation and adoption data

Adoption data was analysed descriptively, and quarterly frequencies are presented graphically. Logged implementation activities were coded independently by two researchers (JJ, CN) against 16 of the 73 ERIC implementation strategies including the 6 core strategies and 10 additional strategies recorded in the implementation activity register [35–37]. A full description of each strategy is outlined in Additional File 2. Some activities included multiple discrete strategies. Any coding discrepancies were discussed with the research team until consensus was reached.

#### Qualitative interviews

Interview transcripts were coded, using a deductive process guided by CFIR, to understand barriers and enablers to adoption with a view to guiding ongoing implementation [38]. Whilst qualitative data were being collected, another member of the research team listened to the interview recordings and read the transcripts, coding and adding notes to familiarise themselves with the data and identify barriers and enablers (JJ). These notes were reviewed, discussed and refined with a second researcher (MD), who had read and coded a pragmatically selected subset of transcripts independently. Team agreement was sought as the determinants were reviewed, refined and reordered. *NVivo12* software *(QSR International Pty Ltd., Melbourne Australia*) was used to support the analysis.

## Results

### Adoption

**Fig. 2:**
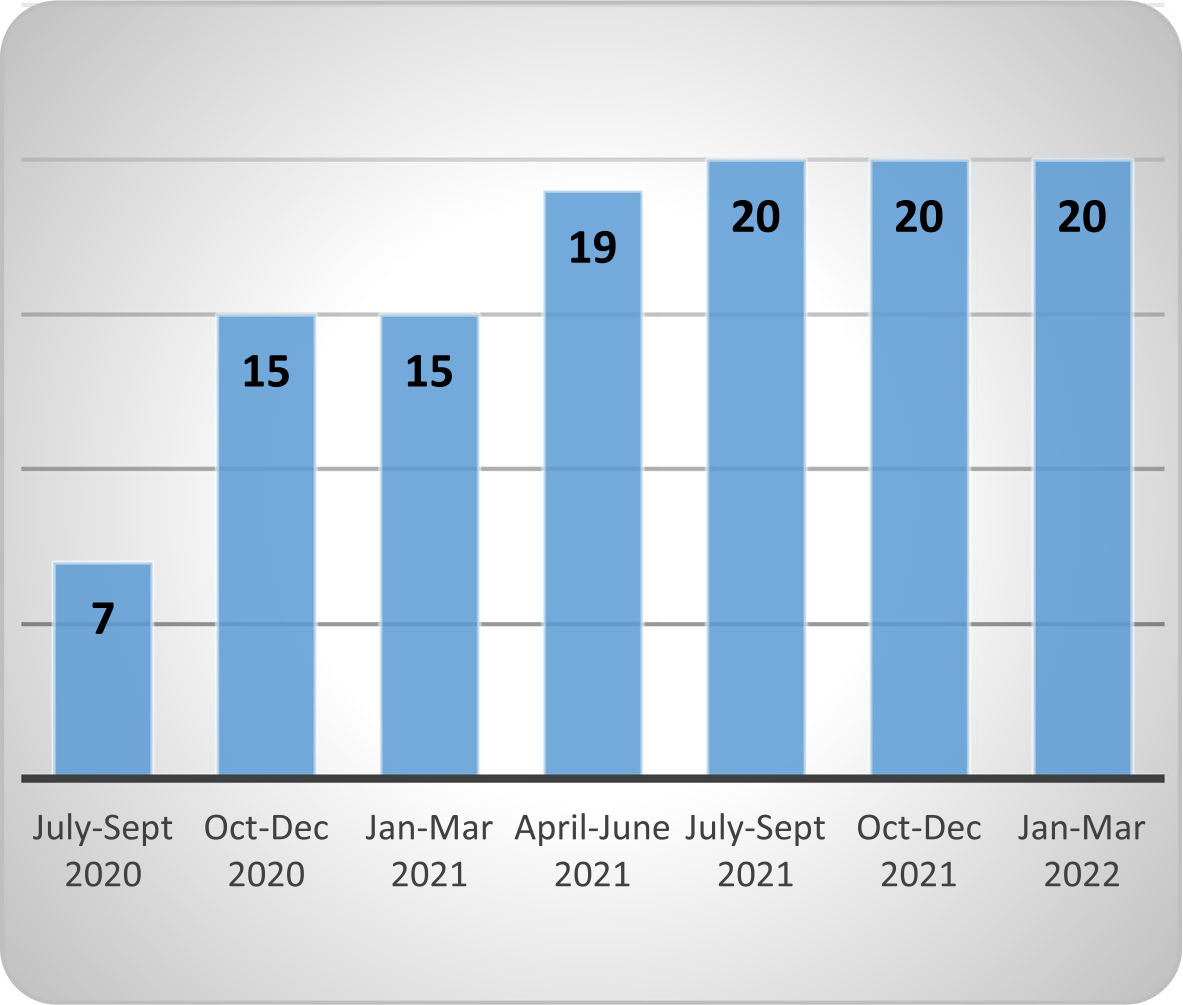
Adoption—number of General Practices enrolled in the service (cumulative)

Of the 40 practices (13 WQPHN, 27 BSPHN) invited to take part in the service, 50% enrolled (20 practices: 11 WQPHN, 9 BSPHN) (Fig. 2). Eight (20%) of the remaining invited practices consented but have not yet completed the enrolment process (one has not yet sent a test message, one did not proceed to installing the template, one practice is progressing the research agreement and five state-operated practices do not have access to the send function of secure messaging). The additional 12 invited practices (30%) have not yet consented (one is temporarily closed, one does not have software linked to secure messaging, and ten are still considering implementation).

**Fig. 3:**
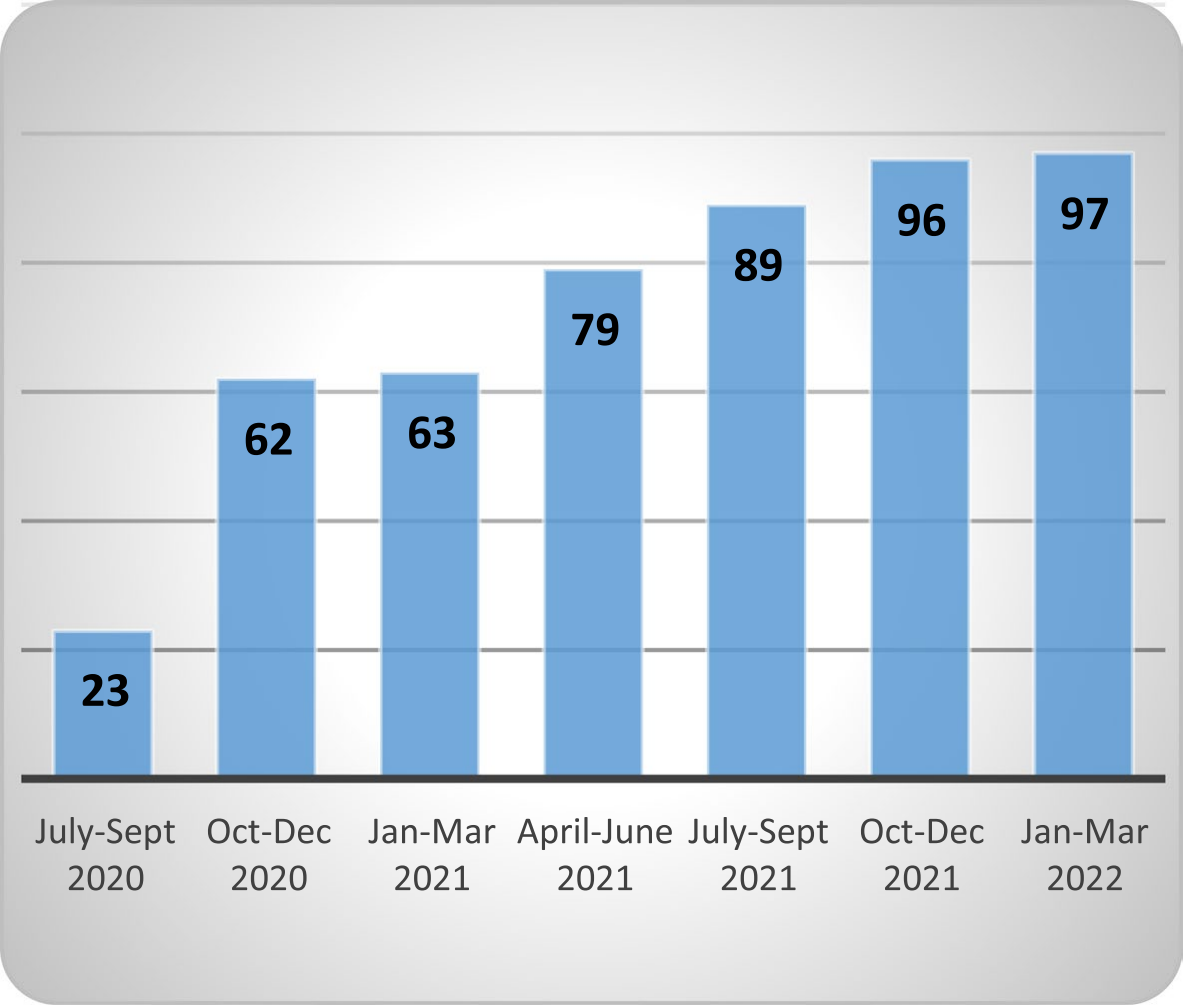
Adoption—number of GPs consented (cumulative)

**Fig. 4:**
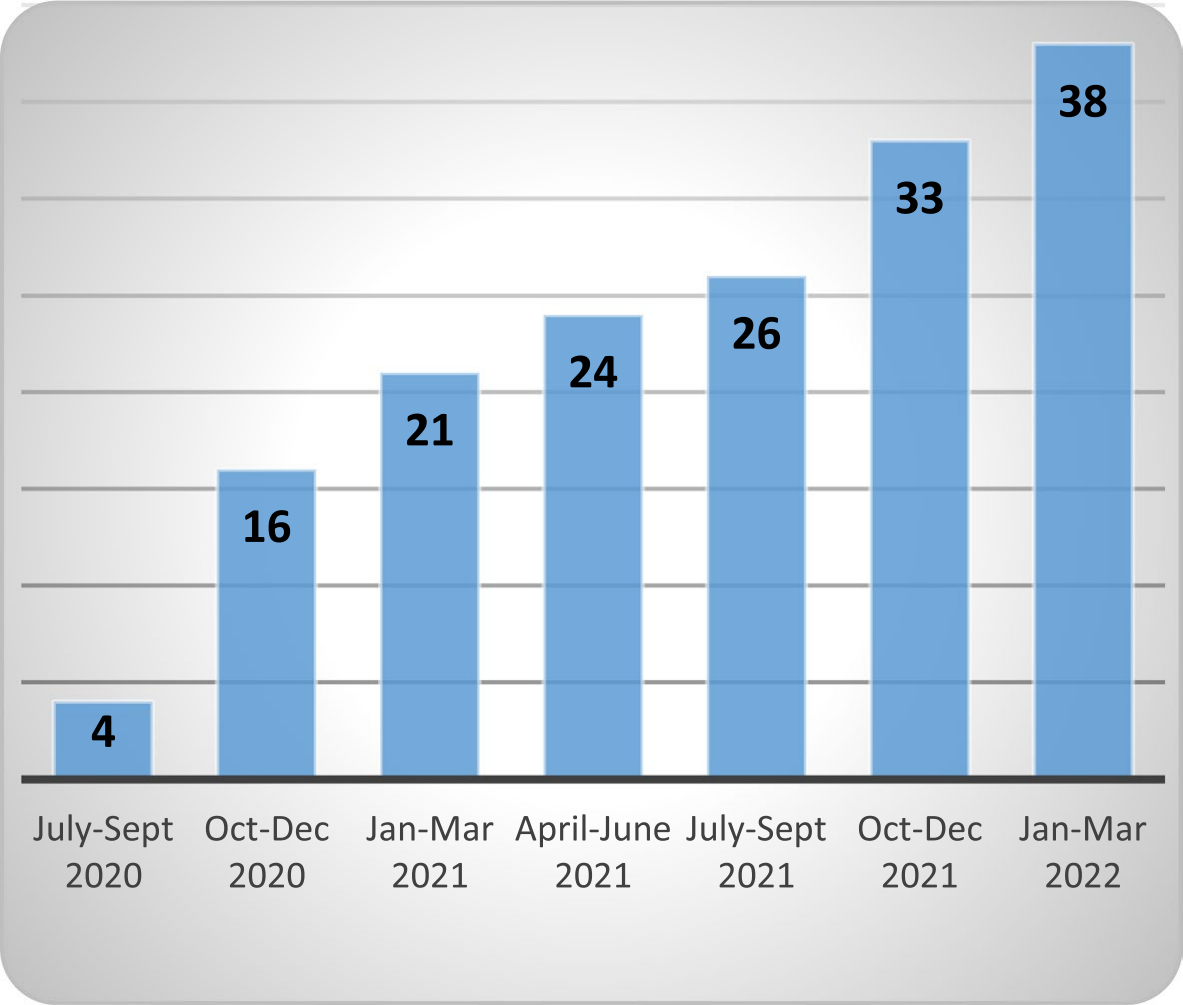
Adoption—number of GPs who use the service (cumulative)

**Fig.5:**
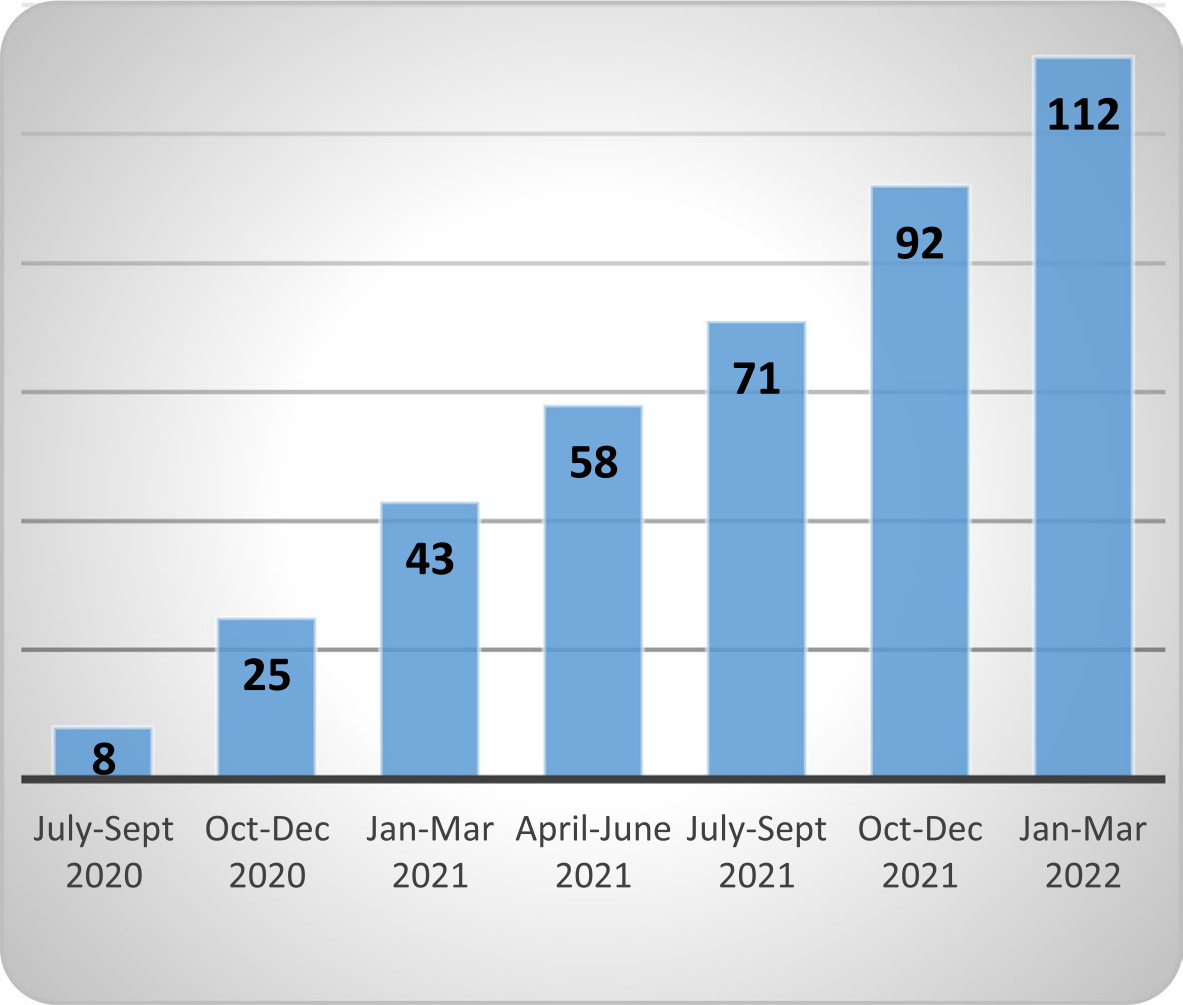
Adoption: Number of RFA sent – (cumulative)

As of March 2022, a total of 97 GPs provided consent (Fig. 3), of which 38 have sent at least one RFA (Fig. 4). During the current phase of implementation (July 2020 – March 2022, i.e., post pilot study) 112 RFAs have been sent (Fig. 5). The number of RFA sent per GP ranged from one to 15 with a median of two. GPs from practices who adopted the service earlier during implementation sent the highest numbers of RFA.

### Implementation activities

**Fig. 6:**
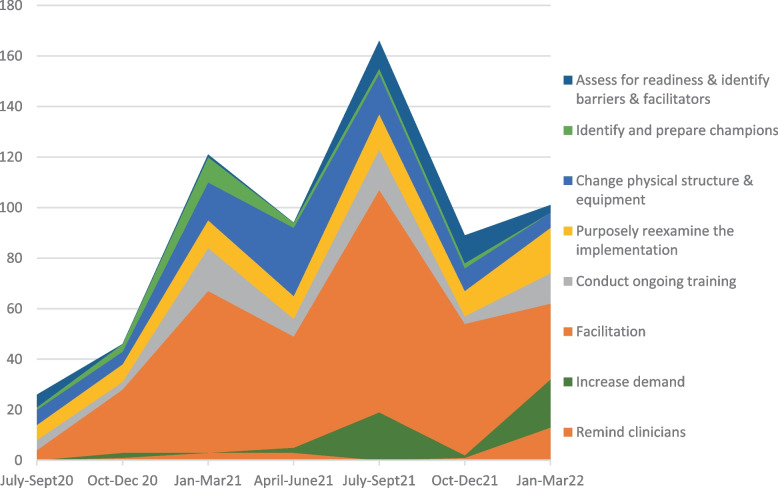
Implementation strategies employed

Between July 2020 and March 2022, 682 implementation activities were recorded. The timing of the eight most frequently used implementation strategies was used to explore temporality (i.e. when the strategy was used), see Fig. 6. Facilitation (support and problem solving) was the most common (48%), followed by managing the changes to the digital technology infrastructure (13%), purposely re-examining the implementation (12%), conducting ongoing training in the use of the service (10%), and increasing demand by informing the broader general practice community about the service (7%).

### Qualitative interviews

Interviews were conducted with 15 stakeholders (11 GPs, 4 other stakeholders). Participants were predominantly female (12/15, 80%) from rural and remote regions (11/15, 73%) and two of the GPs were sole practitioners. Of the 11 GPs, three were high users of the service, five medium users, and one was a low user. Interviews lasted between 12 and 37 min (mean 23 min).

### Enablers

Three overarching eConsultant service enablers were identified including: 1) a perceived advantage of the service over other available options for specialist input, 2) patient benefit, and 3) COVID-19 encouraging uptake of innovative digital solutions.

#### Enabler 1: Relative advantage

Participants emphasised eConsultant is an efficient service, which GPs report as easy to use. Timeliness of the specialist’s response in comparison to traditional outpatient referrals was mentioned by most participants as a key enabler:

*“Getting that response … within 48 hours... is pretty incredible”* (GP-05)

*“By the time eConsultant’s written back to me, that would be the time that the hospital might say I’ve received a* [traditional] *referral, and they’re on a waiting list”.* (GP-03)

eConsultant is viewed as particularly beneficial in rural Queensland where easy access to specialists is limited:

*“They’re usually fly-in, fly-out specialists... they only visit every so often. Sometimes it might be once a month, or as little as once a quarter.”* (SH-04)

GPs described how using eConsultant often results in avoidance of an outpatient referral and highlighted the importance of this in circumstances where they feel the patient cannot wait months or potentially years to be seen be a medical specialist. In some cases, GPs reported using eConsultant meant an outpatient appointment will be more effective because patients have completed the required investigations:

*“If we can do that initial workup using eConsultant can actually save that time.”* (GP-03)

Most GPs felt the process and time to generate a RFA is similar to sending a traditional outpatient referral and appreciated the simple auto (pre)-populated eConsultant template:

*“We’ve got the referral in our medical software… it’s filled out like any other sort of referral… it all gets virtually... pre-populated... so it's not more difficult than any other referral.”* (GP-04)

Many GPs have established practices of seeking advice from more experienced GPs within the practice or specialists who they have an established association with and can be reached by phone. However, the asynchronous nature of communication with the eConsultant service avoids the frustrations of ‘*phone tag*’ that is often required to get specialist advice:

*“If I were to phone for advice, then I'm phoning the switchboard, having to wait... while they try and track down the doctor… you often end up speaking to a registrar who might not be as experienced as a consultant.” (*GP-07).

GPs felt the eConsultant service aligns well with general practice workflows and appreciate that it provides a documented, formalised method for GPs to access specialist advice:

“*Rely*[ing] *on our own network of colleagues that we could ring up and they would just talk to us out of the goodness of their hearts, whereas this is... a more formalised feedback mechanism.”* (GP-03)

The straightforward, implementable advice and actions proffered by the eConsultant were valued by GPs who stressed the importance of having the option for ongoing communication:

*“You get a detailed letter back saying the investigations you should do… which you very rarely get from an outpatient clinic… sometimes you don’t get a letter back at all. You’re relying on the patient.”* (GP-11)

#### Enabler 2: Meeting patient need is highly valued

Patient benefit was viewed almost universally as an enabler of the eConsultant service. GPs valued being able to facilitate patient management in the general practice setting, avoiding significant time and travel costs for patients:

*“Sometimes you have to refer to Cairns, to Townsville, to Brisbane, with all different types of issues.”* (GP-10)

*“The patient can’t get to see a specialist anywhere, because they live too remotely, and I know that they’re not going to travel.”* (GP-02)

*“If I had to refer that patient on, the cost involved for that patient to get to the specialist, and because many of them are a long distance away, and that has a cost involved... because there’s travel, often accommodation, and time for that patient away from work.”* (GP-03)

Patients with multiple comorbidities often require referral to several specialties. GPs felt the eConsultant service offered an efficient alternative for these complex patients:

*“Most of my* [eConsultant] *patients... have been... a bit more complex, a few conditions going on at once, or they’re elderly and frail and don't really want to be going through to multiple consultants… for their various issues... the patients are really grateful not to have to go for unnecessary investigations or outpatient appointments.”* (GP-07)

For these complex patients who may have multiple hospital admissions in a year the ability for the GP to have prompt access to management advice may reduce hospital admissions:

*“it might even avoid a hospital admission, because obviously, if you can answer a question and solve a problem, then that is good for a patient that might be higher risk, and is having two or three hospitalisations a year, just because of the complexity of their disease”* (SH-04)

#### Enabler 3: COVID-19 as an enabler

A reduction in outpatient appointment availability during COVID-19 lockdowns, combined with the increased uptake of other digital solutions, elevated the eConsultant service as a practical and convenient option for specialty input:

*“You just have to get through that thought barrier on how we do business. And I think in this instance, COVID is your friend, because it has made us think about how we can do that. And a lot of us are doing it differently. Yeah, innovation.”* (GP-06)

*“We’ve just all become more comfortable and familiar with doing things on the phone and doing things electronically, like scripts and things so it’s just become the new normal really.”* (GP-11)

### Barriers

Barriers to using the eConsultant service included: 1) variability in digital technology infrastructure, access to digital technology, and digital technology literacy across GPs and practices; 2) competing priorities in a busy practice setting exacerbated by COVID-19; and 3) keeping the service ‘front of mind’ and the ongoing training requirement for rotating staff, locums, and registrars.

#### Barrier 1: Digital technology infrastructure and literacy

GPs and other stakeholders from rural state-operated practices reported barriers related to the status of their clinical information and secure messaging systems:

*“Implementing it is quite difficult because sometimes their software isn’t up to date with the newest version, they may not have the necessary secure messaging version, they might be receiving information, but might not have the ability to send information… to enable some of that we’ve paid for licenses to make sure that people have the necessary technical ability to actually implement the program.”* (SH-04)

GPs in a number of state-operated practices did not have direct access to secure messaging software and were required to take additional steps to send a RFA:

*“It doesn’t communicate directly ... we do the referral as a proof of referring into their chart, but then still have to... copy and paste it over into medical objects... so it's quite clunky, in that respect, and time consuming.”* (GP-05)

For a few GPs, lack of familiarity with digital technology options meant they weren’t as comfortable sending eConsults:

*“Not being an IT guru, I thought that won’t be for me.”* (GP-08)

However, as one stakeholder pointed out this can be overcome quickly given the simplicity of the system:

*“The actual process once they get over that first fear of ‘oh this is an IT program, I don’t know how to do this’, once they’re shown, most of them are actually surprised at how easy it is to do.”* (SH-04)

#### Barrier 2: Competing priorities and implementation climate

Several GPs reported feeling overwhelmed by new initiatives, finding it challenging to implement new programs in addition to managing their current workload:

*“As GPs… you’ve got so many things to think about, so many new programs that they often bring in. So, it’s often like, you learn about it, but you don’t necessarily implement straightaway.”* (GP-07)

COVID-19 has exacerbated this issue, particularly for GPs who have run vaccination clinics:

*“we’ve been vaccinating like crazy… we are exhausted and traumatised… it has been the most extraordinary three years of my life. And that is a barrier because… you just couldn’t do another thing.”* (GP-06)

#### Barrier 3: Keeping eConsultant ‘front of mind’

GPs acknowledged that, as eConsultant was only available for the speciality of general medicine, they did not always remember to use the service. They expressed that they would be more likely to use the service if it was promoted more to keep it “front of mind”, and recognised that in the busy general practice setting this can be challenging, especially as the service is currently limited to one medical specialty:

*“Because I don’t use it that often I keep on forgetting… If it was a broader variety of specialists, then it would come to mind more often.” (GP-02)*

Participants had several suggestions for how uptake of the service could be encouraged. For example, including information on the service in a training package for practice managers, GP locums, and GP registrars when they join a general practice was suggested. This was mentioned particularly for rural practices where there is a reliance on rotating staff, locums, and registrars to fill workforce gaps:

*“Promoted a little bit more so… that doctors are aware of it. So, it’s front of mind” (GP-04)*

*“Improve our orientation for the doctors coming into the practice, the locum doctors and the shorter-term doctors to know that the service is available might improve the usage of it as well.” (GP-03)*

Practice managers are often the conduit to GPs for changes or improvements to programs as GPs are inundated with emails and find it challenging to keep up with required reading particularly with the volume of COVID-19 updates:

*“we are overloaded with our inbox. And we don't often read a lot of material that’s posted to us, unless it’s sort of pointed out to us.. if there’s changes happening with the program, or improvements, using someone like.. (our practice manager), who may be able to bring it to a doctor’s meeting… because we are hopeless at reading things.” (GP-03)*

Including eConsultant case studies in newsletters and as part of the GP training and ongoing education about the service was felt to be important:

*“with the newsletter, case studies that they have dealt with would probably be really useful. So, if I had a similar case, I would go “Oh, yeah, I saw that the eConsultant dealt with this”. Because it is a bit hard to know, what would be suitable for an eConsultant consult. Really, it’s only medication stuff in my kind of assessment” (GP-09)*

Outpatient department correspondence from the hospital to GPs has been suggested as an avenue for reminding busy GPs about the eConsultant service, with GPs reporting they are more likely to read these letters.

*“when a referral goes in to Mater and we get notification back to say they’ve received the referral, they’ve been categorised, and it’ll be three months or whatever COVID permitting... you could just put a little line in there ‘By the way, are you aware, we offer an eConsult for these types of things, there is an option to have an answer within three days’.” (GP-06)*

Additionally, endorsement from fellow GPs, ideally someone with a high profile, and involving influential decision makers as champions of the service were also viewed as potentially useful:

*“Get our directors of medical services to share this information well, and explain what the program benefits are, we will be able to increase our receptivity across the district.” (SH-01)*

## Discussion

Implementation of our eConsultant service in two of Australia’s PHNs has seen a steady increase in adoption and has facilitated consideration of our end-users in developing a blueprint for broader implementation. While the provision of efficient, timely access to specialist support for GPs and their patients are demonstrated primary enablers, two key factors have slowed implementation. Firstly, incompatible digital infrastructure used across the health system was a barrier to implementing the service especially in state-operated general practices; and secondly keeping the service front of mind in the busy general practice setting is a challenge. These issues highlight two strategies to prioritise for successful implementation: (i) a universally accepted digital solution for the eConsultant service, and (ii) an effective engagement approach.

As described by others, adoption of our eConsultant service is enabled by alignment with GPs’ usual referral practices [39]. Most general practices employ secure messaging software linked to clinical information systems which maximises the ability to auto-populate the RFA with patient information before sending. However digital infrastructure varies greatly across general practices in rural Queensland. Whilst most private general practices use a secure messaging provider able to transfer pathology, radiology and other clinical information readily, other practices were without this support. Where a private general practice did not have secure messaging, our organisational partner, the WQPHN, funded licensing for the first year. This however, was not possible in state government-operated practices, which generally operate under health department business regulations. For many of these practices, absence of the secure messaging ‘send’ function, prohibited adoption of the eConsultant service. General practice adoption of other electronic referral processes in Queensland has been met with similar barriers. A standardised messaging solution and interoperability between digital systems is recognised by GPs and stakeholders as key to facilitating adoption of broader clinical linkage including eConsultant [27, 40].

In addition to a universal digital solution, expanding to other specialties will increase the opportunities for GPs to use the service more regularly, keeping the service ‘front of mind’ and increasing the likelihood of it as the first option in appropriate clinical situations. This was so in Canada, where Liddy and colleagues found more rapid adoption during implementation of the service when additional medical specialties were available [41]. Planning early for sustainability has been proposed as a key element to implementation of eConsultant and adequate eConsult funding for specialist time to respond to RFA will support expansion of the program to additional specialties [42, 43].

Our implementation evaluation has highlighted strategies to promote ongoing engagement with eConsultant including identifying GP and practice champions [23, 44]; facilitating support from Directors of Medical Services; and regular webinars and newsletters for GPs with eConsultant case studies. Case study webinars offer an opportunity to build the relationship between GPs and specialists which others have reported to be an enabler to eConsultant implementation [45]. For practices, particularly in remote and rural Queensland, the workforce is often GP locums, GP registrars or GPs who rotate from week to week, with some GPs having a two to three-month break between blocks. Bundling eConsultant training into orientation packages for new and locum staff will increase awareness in these areas, provide staff with an efficient advice option, and support ongoing implementation of the eConsultant service.

Supported by international literature, this evaluation has highlighted that those GPs and practices who have been early adopters of eConsultant can see the relative advantage to patients of improved access to specialist input without the need for travel and long wait times, and these GPs find it easy to use and compatible with their existing workflows [20]. Once GPs have sent their first RFA they report it being a relatively simple process and importantly, they are experiencing education benefits and improved patient management. Stakeholders value the benefits to the health system of timely specialist support for patients without the cost to the health system of subsidised patient travel, face-to-face visits, and potentially preventable hospital admissions.

### Strengths and limitations

While COVID-19 management and vaccination has placed excessive burden on the general practice system which has slowed our eConsultant implementation and evaluation, it has highlighted the importance of providing structured remote access for GPs and patients to specialist support, and of reducing in person hospital visits for OPD appointments. Using the IRLM to integrate theory underpinning our implementation has allowed us to examine and modify our strategy bundle for ongoing implementation. Continuing quality and safety monitoring informs our service and fidelity outcomes which have been previously reported [22]. The next step in our research as implementation expands is to evaluate sustainability and service outcomes including a costing analysis to inform the benefits from both the patient and health system perspective. There are limitations to our evaluation. Non-users of the eConsultant service were not interviewed meaning there may be additional barriers to use that remain unknown. Also, to facilitate low participant data collection burden, implementation activity tracking included those activities conducted jointly by the research team and organisational partners but does not include additional activities undertaken by our partners alone.

## Conclusion

This study supports international findings [23, 46] that a commitment to continuous service evaluation is central to strengthening implementation of an ongoing eConsultant service. eConsultant is implementable in the Australian general practice context and offers a streamlined evidence-based option for patients and GPs to access timely specialist input. A well-integrated digital system and expanding to additional medical specialities are essential for broader adoption and translation of the eConsultant service. 

## Supplementary Information

**Additional file 1.** Interview Guide for GPs andStakeholders – Identifying the enablers and barriers to using eConsultant.

**Additional file 2:** **Table 1.** Implementationstrategy definitions and description of activities.

## Data Availability

The adoption and tracking datasets generated and analysed during the current study are not publicly available as the data is not in a de-identifiable format that is appropriate for public distribution. The de-identified transcripts from the interviews are not suitable for public distribution but are available from the corresponding author on reasonable request.

## Abbreviations

BSPHN: Brisbane South Primary Health Network
CFIR: The Consolidated Framework for Implementation Research
eConsultant: Electronic consultation
ERIC: Expert Recommendations for Implementing Change
IRLM: Implementation Research Logic Model
RFA: Request for Advice
WQ: Western Queensland Primary Health Network
