# Supplementary material for: Implementing a general practitioner-to-general physician eConsult service (eConsultant) in Australia
Source: BMC Health Serv Res. 2022 Oct 24;22:1278. doi: 10.1186/s12913-022-08663-2 (PMC9589630; doi:10.1186/s12913-022-08663-2)
Supplement: Supplementary file 1 — Additional file 1. Interview Guide for GPs andStakeholders – Identifying the enablers and barriers to using eConsultant. [file 12913_2022_8663_MOESM1_ESM.docx]

**Interview Guide for GPs – Identifying the enablers and barriers to using eConsultant**

**Interviewer Introduction**

Thanks for agreeing to take part we really appreciate you taking the time to tell us your thoughts about the eConsultant program.

As explained on the Information Sheet, I’ll be recording the interview so I have an accurate record of what was said, but the recording and the transcript will be de-identified and no name linked data will be reported.

Do you have any questions you’d like to ask before I start recording?

**Global Question**

1. I’d like to start by hearing your general thoughts about eConsultant and your experience using it?

**Intervention Characteristics**

1. What factors play a role in your decision about whether or not to use eConsultant with eligible patients?
2. How does eConsultant compare to a traditional outpatient referral?
3. How complicated do you find eConsultant to use, for example how well does it fit with your existing work processes? (Prompts: number of steps involved, time to prepare the Request for Advice; talking to your patients about the option)
4. In your opinion, how could eConsulant be improved?
5. *Brisbane South only:* Do you use any other electronic referral services and if so how does eConsultant compare with them? (prompts: what advantages does eConsultant have compared to existing options?)

**Patient Needs & Resources**

1. In what ways does eConsultant meets the needs of patients?
2. How have your patients responded to the eConsultant option?
3. In your opinion what proportion of the patients you’ve referred to eConsultant would you have otherwise referred to a Specialist Outpatient Department?

**Cost**

1. What are the key costs relevant to you in undertaking eConsultant?

**Implementation Climate**

1. Who do you believe are the key influential individuals and organisations to get on board with implementing eConsultant? (prompt: both within your practice and externally; are there people in your practice who are champions for eConsultant)
2. What other high-priority activities are happening in your practice and how have these impacted your use of eConsultant? (competing priorities)
3. To what extent has your practice set goals for implementing eConsultant? (Prompts: what are the goals? how are the goals communicated? to whom are they communicated?)

**Design Quality & Packaging and Access to Knowledge & Information**

1. What supports and resources are available to help you use eConsultant?
2. What is your perception of the quality of the supporting materials for eConsultant? Why?
3. Do you believe you received sufficient training for eConsultant? (prompts: What are the positive aspects of training? What is missing?)
4. Have you received and read the eConsultant newsletters? (prompts: how helpful are they? How could they be improved?)

**Close**

Finally, do you have anything else you’d like to add?

Again, thank you for taking the time to participate in the interview, a Coles e-Gift card will be emailed to you to in the coming days (except State employees).

**Interview Guide for Stakeholders – Identifying the enablers and barriers to using eConsultant**

**Interviewer Introduction**

Thanks for agreeing to take part we really appreciate you taking the time to tell us your thoughts about the eConsultant program.

As explained on the Information Sheet, I’ll be recording the interview so I have an accurate record of what was said, but the recording and the transcript will be de-identified and no name linked data will be reported.

Do you have any questions you’d like to ask before I start recording?

**Global Question**

1. I’d like to start by hearing your general thoughts about eConsultant?

**Intervention Characteristics**

1. What factors do you believe could influence whether GP Practices decide to implement eConsultant?
2. How do you see eConsultant being used by GPs (traditional outpatient referral)?
3. How complicated do GP practices find eConsultant to set-up and implement?
4. In your opinion, how could eConsulant be improved?
5. Do Practices in your region use any other electronic referral services and if so how does eConsultant compare with them? (prompts: what advantages does eConsultant have compared to existing options?)

**Patient Needs & Resources**

1. In what ways does eConsultant meets the needs of patients?

**Cost**

1. What are the key costs relevant to implementing eConsultant?

**Implementation Climate**

1. Is there a strong need for eConsultant? Why or why not?
2. What is the general level of receptivity in your organization to implementing eConsultant? Why?
3. Who do you believe are the key influential individuals and organisations to get on board with implementing eConsultant? (prompt: are there people who are champions for eConsultant)
4. What kind of infrastructure changes are needed to implement eConsultant? (Changes in formal policies? Changes in IT or electronic records systems? Other?)
5. What kind of approvals are needed? Who needs to be involved?
6. What other high-priority activities are happening in GP practices in your region and how have these impacted use of eConsultant? (competing priorities)

**Close**

Finally, do you have anything else you’d like to add?

Again, thank you for taking the time to participate in the interview, a Coles e-Gift card will be emailed to you to in the coming days (except State employees).
