# Supplementary material for: Implementing a general practitioner-to-general physician eConsult service (eConsultant) in Australia
Source: BMC Health Serv Res. 2022 Oct 24;22:1278. doi: 10.1186/s12913-022-08663-2 (PMC9589630; doi:10.1186/s12913-022-08663-2)
Supplement: Supplementary file 2 — Additional file 2: Table 1. Implementationstrategy definitions and description of activities. [file 12913_2022_8663_MOESM2_ESM.docx]

**Additional File 2:** Table 1: Implementation strategy definitions and description of activities

| Strategy | Definition^1^ | Description of activities |
| --- | --- | --- |
| 1.Assess for readiness and identify barriers & facilitators | Assess various aspects of an organization to determine its degree of readiness to implement, barriers that may impede implementation, and strengths that can be used in the implementation effort. | Discussion with PHN representatives, general practice managers. Orientation meetings with general practice staff. |
| 2. Build a coalition | Recruit and cultivate relationships with partners in the implementation effort. | Partners operational meetings (1-2 monthly), Queensland eConsultant Partnership steering committee meetings (1-2 yearly), Mater facilitation. |
| 3.Change physical structure and equipment | Evaluate current configurations and adapt, as needed, the equipment to best accommodate the targeted innovation. | Establishing the secure messaging facility (medical objects) where not already available. Installation of eConsultant templates in practice software. |
| 4.Conduct local consensus discussions | Include local providers and other stakeholders in discussions that address whether the chosen problem is important and whether the clinical innovation to address it is appropriate. | Meetings and email/telephone communication with stakeholders. |
| 5. Conduct local needs assessment | Collect and analyse data related to the need for the innovation. | WQPHN/BSPHN needs assessment. |
| 6. Conduct ongoing training^a^ | Plan for and conduct training in the clinical innovation in an ongoing way. | Emailing and posting instruction manuals/in-person and zoom training sessions/webinars/site visits/ cheat sheets/newsletters (bi- monthly). |
| 7. Develop and implement tools for quality monitoring | Develop, test, and introduce into quality-monitoring systems the right input—the appropriate language, protocols, algorithms, standards, and measures (of processes, patient/consumer outcomes, and implementation outcomes) that are often specific to the innovation being implemented. | Develop and implement audit tools, introduce a close-out survey, distribute and collate GP and general physician data collection sheets. |
| 8. Facilitate relay of clinical data to providers | Provide as close to real-time data as possible about key measures of process/outcomes using integrated modes/channels of communication in a way that promotes use of the targeted innovation | Webinars, publication, conference presentations of eConsultant service implementation evaluation outcomes. |
| 9. Facilitation^a^ | A process of interactive problem solving and support that occurs in a context of a recognized need for improvement and a supportive interpersonal relationship. | Phone/ email/ in-person support in using the service. |
| 10. Identify and prepare champions | Identify and prepare individuals who dedicate themselves to supporting, marketing, and driving through an implementation, overcoming indifference or resistance that the intervention may provoke in an organization. | Site visits, phone calls, and training sessions used to identify and prepare champions to support and drive eConsultant. |
| 11. Identify early adopters | Identify early adopters at the local site to learn from their experiences with the practice innovation. | Site visits, phone calls, and training sessions used to identify and early adopters of eConsultant. |
| 12. Increase demand | Attempt to influence the market for the clinical innovation to increase the maturity of the market for the clinical innovation. | Distribution of eConsultant newsletters to stakeholders for circulation, adding eConsultant to BS and WQ Health Pathways website, presentations to Health and Hospital Service groups (Queensland Health), GP Liaison Office groups and GP registrar training groups. |
| 13. Provide local technical assistance | Develop and use a system to deliver technical assistance focused on implementation issues using local personnel. | IT support via phone and email for using the service. |
| 14. Purposely re-examine the implementation | Monitor progress and implementation strategies to continuously improve the quality of care. | Fortnightly team implementation meetings/ 1-2 monthly meetings with stakeholders. |
| 15. Remind clinicians | Develop reminder systems designed to help clinicians to recall information and/or prompt them to use the clinical innovation. | Newsletters, emails and phone calls, webinars and practice visits to remind GPs about the service. |
| 16. Stage implementation scale up | Phase implementation efforts by starting with small pilots or demonstration projects and gradually moving to a system wide rollout. | Proof-of-concept (one urban general practice), pilot study (four rural/remote general practices), implementation expanded in stages to additional WQ and BS general practices. |

^a^ Training and facilitation were generally conducted via phone and zoom except for seven BSPHN Practices which each received one in-person visit.

1. Powell BJ, Waltz TJ, Chinman MJ, et al. A refined compilation of implementation strategies: results from the Expert Recommendations for Implementing Change (ERIC) project. *Implementation Science* 2015;10(1):21. doi: 10.1186/s13012-015-0209-1
